# Learning Curve of Thoracoscopic Repair of Esophageal Atresia

**DOI:** 10.1007/s00268-012-1651-8

**Published:** 2012-05-15

**Authors:** David C. van der Zee, Stefaan H. A. J. Tytgat, Sander Zwaveling, Maud Y. A. van Herwaarden, Daisy Vieira-Travassos

**Affiliations:** Department of Pediatric Surgery KE.04.140.5, Wilhelmina Children’s Hospital, University Medical Centre Utrecht, P. O. Box 85090, 3508 AB Utrecht, The Netherlands

## Abstract

**Background:**

Thoracoscopic repair of esophageal atresia is considered to be one of the more advanced pediatric surgical procedures, and it undoubtedly has a learning curve. This is a single-center study that was designed to determine the learning curve of thoracoscopic repair of esophageal atresia.

**Methods:**

The study involved comparison of the first and second five-year outcomes of thoracoscopic esophageal atresia repair.

**Results:**

The demographics of the two groups were comparable. There was a remarkable reduction of postoperative leakage or stenosis, and recurrence of fistulae, in spite of the fact that nowadays the procedure is mainly performed by young staff members and fellows.

**Conclusions:**

There is a considerable learning curve for thoracoscopic repair of esophageal atresia. Centers with the ambition to start up a program for thoracoscopic repair of esophageal atresia should do so with the guidance of experienced centers.

## Introduction

It has been 10 years since the first feasibility studies on thoracoscopic repair of esophageal atresia in neonates were first published [1–3]. Since then, the thoracoscopic approach has become more widespread [4–8]. Recently, in a British survey 46 % of the participating pediatric surgeons indicated that they were intending to start with the thoracoscopic approach for esophageal atresia repair [9].

Thoracoscopic repair of esophageal atresia is considered to be one of the more advanced pediatric surgical procedures. What is the learning curve of this procedure?

During the first five years the thoracoscopic repair of esophageal atresia was mainly performed by the senior pediatric surgeons, whereas during the second five years the procedure was mainly performed by younger staff members and fellows under supervision of the senior staff.

This is a single-center study comparing the first five years of thoracoscopic repair of esophageal atresia to the second five years in which this procedure was employed.

## Materials and methods

**Fig. 1:**
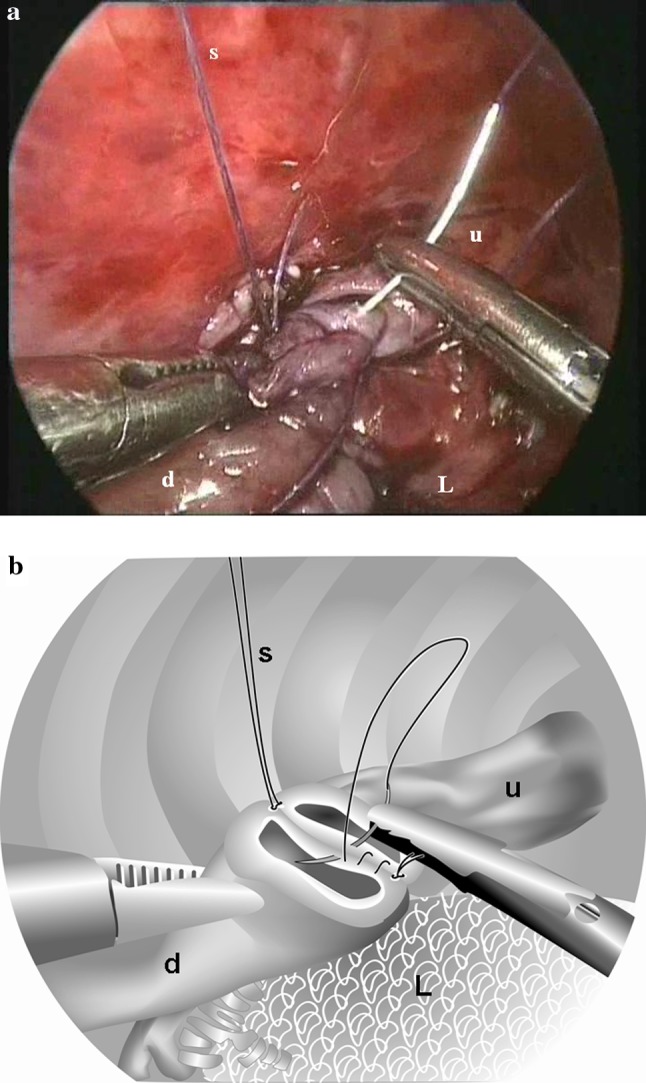
Running suture of posterior wall. Upper esophagus (*u*), distal esophagus (*d*), and lung (*L*). A stay suture (*S*) stabilizes the esophagus for suturing. **b** Drawing of the picture in **a** schematically displaying the posterior wall of the upper (*u*) and distal (*d*) esophagus, where a running suture has been laid. This running suture is tied to the stay suture (*S*) laid at the posterior wall. The collapsed lung (*L*) is at the lower end

**Fig. 2:**
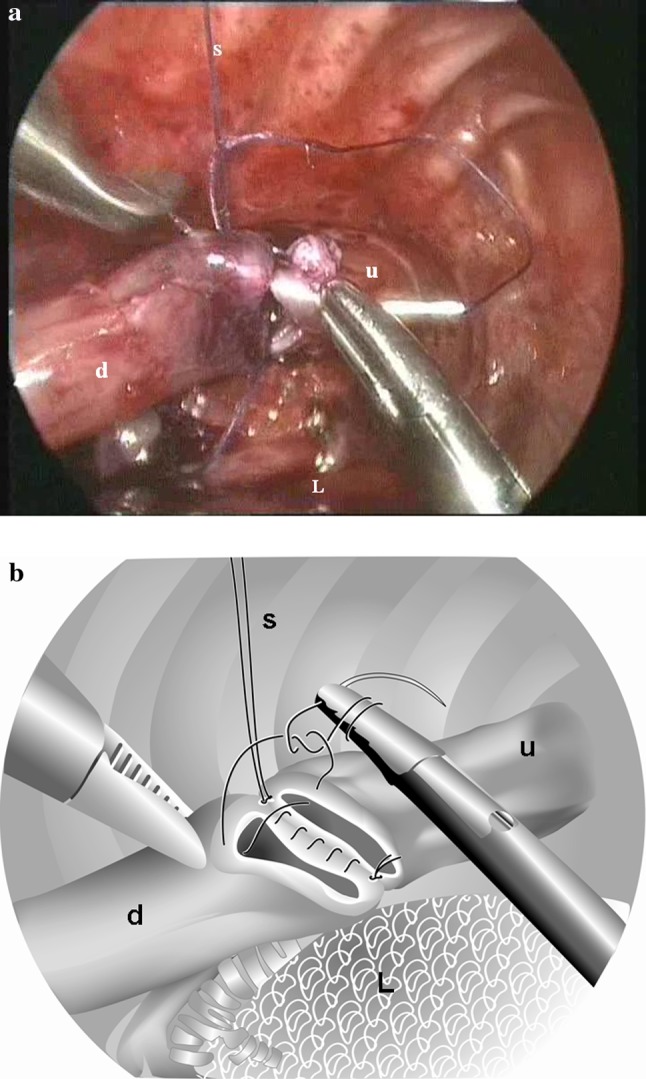
Start of a running suture along the anterior side of the esophagus (this can be the same suture used for the posterior wall after it has been tied to the stay suture). Upper esophagus (*u*), distal esophagus (*d*), and lung (*L*), as well as the stay suture (*S*), are marked. **b** Drawing of the picture in **a** demonstrating the start of the anterior running suture

The operative technique for thoracoscopic correction of esophageal atresia has been extensively described elsewhere [10]. Here only the refinements, that in our opinion have led to further reduction of complications, will be explicited. The first important factor is the ligation of the fistula with a transfixing Vicryl 3 × 0 ligature, that will not come off later during any event. A second important factor is the use of a stabilizing first suture on the back wall that is drawn out through the thoracic wall and fixed with a mosquito clip (Fig. 1a, b). This will make maneuvering easier without crushing the tissue with forceps. The third factor is the use of a running suture whenever possible, because this will secure a watertight anastomosis, where sometimes the space between two interrupted sutures may be too big and lead to leakage (Fig. 2a, b). Finally, the experience of the senior surgeons, who always assist in the procedures, will avoid the pitfalls usually made by beginning teams. When the primary surgeon tends to make the wrong decision, the senior surgeon intervenes and puts the primary surgeon back on the right track. Principally, no chest tube is left behind.

Early postoperative follow-up is based on clinical symptomatology. The child usually is extubated the following day or the second day, and feeding through the transanastomotic tube is started. When there is no more saliva retention, oral feeding can be started on day 3–5. Only when the patient develops a temperature or is experiencing pulmonary compromise is a thoracic X-ray made. If there is effusion in the right thoracic cavity, a sign of anastomotic leakage, a thoracic drain is placed through one of the trocar incisions for a few days until the leak dries up. Only when doubt persists is a contrast study performed. Also in the further follow-up, contrast studies are only performed when symptoms of stenosis, such as swallowing difficulties (i.e., feeding difficulties) arise. All children with esophageal atresia receive ranitidine during the first month of life. In case of stenosis, balloon dilatation is carried out under general anesthesia. If the stenosis persists or recurs too often, a laparoscopic antireflux operation may be considered.

Between May 2000 and December 2010 a total of 72 patients with type C esophageal atresia underwent thoracoscopic repair in our department. This time period was split into two segments: 2000–2005 and 2006–2010. Demographic data, co-morbidity, operative data, and postoperative follow-up were compared.

Statistical analysis was performed with the Wilcoxon rank sum test for continuous variables. For categorical variables the chi-square test and Fisher’s exact test were used.

## Results

The demographics are displayed in Table 1. There were 41 consecutive patients with type C esophageal atresia in the first period and 31 consecutive patients in the second. Both populations were comparable as to gestational age, birth weight, and associated anomalies.

**Table 1:** Demographics of patients with esophageal atresia

Demographics	2000–2005	2006–2010
No. patients	41	31
Gestational age (weeks)	37.5/7	37.4/7
Birth weight (g)	2,660	2,620
Associated anomalies	28 (68 %)	16 (51 %)

The outcome of surgery is displayed in Table 2. The smallest child was born after 34.3/7 weeks of gestation and weighed 1,025 g, indicating that premature and low-birth-weight neonates are principally approached thoracoscopically. There was no difference in operating time between the first and second time periods. Conversion was necessary in two patients in the period 2000–2005. In the first child, that occurred early in our experience when only 5 mm trocars were available in our hospital; low birth weight and small size required conversion to prevent damage to the ribs. The second patient did not tolerate CO_2_ insufflation and later was diagnosed with an undetected atrial septal defect (ASD) type II requiring cardiac surgery. Median admission time in the ICU and hospital were not significantly different.

**Table 2:** Postoperative outcome in two time periods, 2000–2005 and 2006–2010

Operative results	2000–2005	2006–2010	*P* value
Median operating time (min)	155	160	
Conversion	2	2 (long gap)	
Median IC admission (days)	4	4	
Median feeding time (days)	4.56	4.25	
Median admission time (days)	16.5	14.3	
Postoperative leakage	9 (22 %)	2 (8 %)	0.082
Recurrent fistula	2 (4 %)	0	
Postoperative stenosis	16 (38 %)	6 (19 %)	0.062
Postoperative death	1	1	

Postoperative leakage decreased markedly in the period 2005–2010 (22 to ≥8 %). The incidence of postoperative stenosis had diminished from 16 to 6 patients (38 to ≥19 %). Recurrent fistulae were not present in the second time period. In both time periods there had been one death. The first was a child with Feingold syndrome whose parents refused further treatment. The second child, a twin, was born extremely prematurely and was dysmature at a birth weight of 830 g at 31.6/7 weeks, and had a tracheal rupture during surgery.

## Discussion

Thoracoscopic repair of esophageal atresia is considered to be one of the more advanced endoscopic procedures in pediatric surgery [5, 7, 8]. For this type of advanced procedure, a learning curve has to be taken into account [8]. The thoracoscopic repair of esophageal atresia in our department has been performed for 10 years now. In the present study, a comparison was made between the first and second five-year time periods to determine if there had been a change in outcome, reflecting a learning curve. This learning curve could then be considered by other centers that are planning to start a program for thoracoscopic repair of esophageal atresia.

The patients treated in both five-year periods were comparable in terms of gestational age, birth weight, and associated anomalies.

The duration of operation remained unchanged. This can be explained by the fact that in the second time period other staff members and fellows principally performed the procedure under supervision of one of the senior pediatric surgeons. The operating surgeons could thus practice the procedure at their own speed, but with the parachute of the supervising senior surgeon to guarantee quality control. This is an issue for centers that wish to start up the thoracoscopic approach for correction of esophageal atresia. Because of the considerable learning curve, it is advisable for less experienced surgeons to perform the procedure under the guidance of an experienced endoscopic surgeon.

The length of postoperative stay in the intensive care unit did not seem to lessen in the second five-year time period. This was likely due to an unchanged, conservative postoperative treatment protocol used in the ICU. Ceelie et al. [11] had concluded in their study that endoscopic surgery had no effect on postoperative pain management. However, they had failed to adjust their pain protocol to the new approach, resulting in a similar outcome as before. These findings have led to alterations in our current postoperative treatment protocol for patients who have undergone thoracoscopic esophageal atresia repair. Patients will now only receive morphine based on their pain score. This may make it possible for the patients to be weaned from the ventilator earlier.

The time to first feeding had also not changed for the same reasons as mentioned above. This too has now been changed in the postoperative treatment protocol. Feeding through the transanastomotic tube will be started on the second postoperative day, and oral feeding will be commenced as soon as the child displays no more excessive saliva production. We believe that this may ultimately result in a shorter hospital stay.

The digital measurement in the Picture Archiving and Communication System (PACS) of the length of the proximal esophagus and distance to the carina are good predictors for risk of postoperative leakage in children with esophageal atresia and distal fistula, because of the more or less fixed position of the esophagus and trachea in the thoracic cage [12]. In this study we demonstrated that patients with a proximal esophagus measuring less than 7 mm, and a distance from the carina to the proximal esophagus of more than 13.5 mm, have an increased risk for postoperative leakage after primary repair of their esophageal atresia. We have used this technique since and operated on an additional 20 children. Of these children, 5 fulfilled the criteria described above, but only 2 indeed had postoperative leakage.

Overall there was a remarkable reduction in postoperative leakage from 22 to 8 %. Although the difference was not significant due to the small numbers, we believe that increased surgical expertise and the technical adjustments (Figs. 1, 2) led to this reduction in postoperative leakage. Summarizing, these adjustments were (1) ligation of the fistula with a transfixing Vicryl 3 × 0 ligature; (2) use of a stabilizing first suture; (3) use of a running suture; (4) supervision by an experienced surgeon. The reduction of postoperative leakage in our study was similar to that observed in patients who had undergone laparoscopic repair of their duodenal atresia. The use of traction sutures and incorporating running sutures resulted in a more watertight anastomosis [13]. A similar report on technique was published by Shimotakahara et al. [14]. The fact that nowadays young colleagues and fellows perform the operation did not increase the leakage rate. Other endoscopic series describe a leakage rate of 11.5 to 27 % [4, 7, 15, 16].

In the initial period two patients had a recurrent fistula. Since the use of transfixing sutures to close the fistula was begun, no more recurrences have been observed. Other series describe 1.9 to 4 % recurrent fistulae [4, 15].

The incidence of postoperative stenosis had also decreased, from 38 to 19 %. It is not clear if this was due to the operative technique and/or to increased experience. However, we know that in the beginning of implementing the thoracoscopic approach the magnification of the image resulted in performing a too small an incision in the proximal esophagus. Stricture formation or stenosis occurred in 14 to 34.6 % in other series [4, 7, 15, 16].

The thoracoscopic repair of esophageal atresia remains a challenge for many pediatric surgeons. Nevertheless in a British survey [9] 46 % of the pediatric surgeons have indicated that they would like to start implementing this procedure. It is obvious that it will be important to know the pitfalls and the techniques to perform a safe anastomosis. As this study has demonstrated, there is a considerable learning curve, but under guidance of experienced pediatric endoscopic surgeons an extended learning curve can be avoided. Centers with the ambition to start up a program for thoracoscopic repair of esophageal atresia should do so with guidance of experienced centers.

Overall the outcome of thoracoscopic repair of esophageal atresia can very well compete with the outcome of open esophageal atresia repair found in the literature [5, 8, 15]. The additional advantages of the endoscopic approach are better cosmesis and avoidance of possible thoracic wall deformities. Newly adjusted protocols for thoracoscopic esophageal atresia repair in the future will further have to demonstrate their advantage over open surgery in relation to postoperative complications, ICU and admission time and long-term sequelae, such as esophageal stenosis.
